# CSRP1 expression is associated with a mesenchymal, stroma-rich tumor profile and poor prognosis in colon cancer

**DOI:** 10.55730/1300-0144.5736

**Published:** 2023-10-31

**Authors:** Seçil DEMİRKOL CANLI

**Affiliations:** Division of Tumor Pathology, Department of Clinical Oncology, Cancer Institute, Hacettepe University, Ankara, Turkiye

**Keywords:** Colon cancer, bioinformatics, gene expression, CSRP1, prognosis

## Abstract

**Background/aim:**

Cysteine and glycine-rich protein 1 (CSRP1) is involved in the cysteine-rich protein family and is a marker of smooth muscle lineages. In colon cancer, the expression of this gene is associated with poor prognosis. In this study, the aim was to reevaluate its prognostic relationship in independent cohorts and explore potential underlying biological processes that are linked to aggressive behavior in tumors with high CSRP1 expression, such as epithelial-to-mesenchymal transition (EMT), stromal fractions in the tumor microenvironment, and consensus molecular subtypes (CMSs).

**Materials and methods:**

RNA sequencing (RNAseq)-, microarray-, and single-cell RNAseq (scRNAseq)-based transcriptomic data were obtained from public databases. The EMT score was calculated based on the expression of E-cadherin and vimentin genes using a previously published method. The stromal score generated by the ESTIMATE method was utilized for the analysis of correlation with the CSRP1 expression. The scRNAseq data were analyzed via the Seurat R package. The immunohistochemistry-based protein level expression of CSRP1 was evaluated using the Human Protein Atlas database.

**Results:**

Lower CSRP1 expression was noted in colon tumors compared to normal colon tissue. Patients with a high CSRP1 expression had shorter recurrence-free, overall, and disease-specific survivals in the GSE39582 and GSE17536 datasets (p < 0.05). The methylation level of the CSRP1 gene was negatively correlated (r = −0.57, p < 0.0001) with CSRP1 expression in The Cancer Genome Atlas colon adenocarcinoma dataset. CSRP1 expression was positively correlated with the expression of mesenchymal markers, EMT score, and stromal score obtained via the ESTIMATE method. CMS4 colon tumors had a significantly higher CSRP1 expression compared to other CMSs. Analysis of the scRNAseq data revealed that CSRP1 was expressed by epithelial cells and cancer-associated fibroblasts in the colorectal tumor microenvironment, which was also confirmed by the protein expression data from the Human Protein Atlas database.

**Conclusion:**

CSRP1 expression is associated with CMS4, a more mesenchymal stroma-rich molecular profile, and poor prognosis in colon cancer.

## 1. Introduction

Cysteine and glycine-rich protein 1 (CSRP1), a member of the cysteine-rich protein family, is a LIM domain-containing protein involved in smooth muscle differentiation [1]. It is considered as a well-known marker for smooth muscle lineages [2]. CSRP1 has a role in actin filament bundling by cross-linking actin filaments and stabilizing the interaction of alpha-actinin with actin filament bundles [3]. In healthy tissues, the highest transcript level of CSRP1 was reported in the prostate and colon, followed by the brain and testis [4]. In human skin fibroblasts, CSRP1 expression was induced by growth-inhibitory signals, and was associated with a differentiated morphology towards the myofibroblast lineage [5]. The ectopic expression of CSRP1 in these cells was reported to suppress cell proliferation [5]. Supporting these findings, another study showed that transforming growth factor-beta (TGF-β1) increased the expression of CSRP1 at the protein level concomitant with an increase in myofibroblast differentiation [6]. Higher CSRP1 expression was also reported in lung fibrosis compared to control lung tissue [6].

However, relatively little is known about its function and potential implications in cancer. In hepatocellular carcinoma (HCC), 56% of cases had aberrant methylation and a lower expression of CSRP1 compared to normal liver tissue [7]. CSRP1 was listed among hub genes that are downregulated in prostate cancer compared to benign prostate hyperplasia [8]. Moreover, high CSRP1 expression was associated with longer disease-free survival (DFS) in prostate cancer [8]. In colon cancer, CSRP1 has been listed in a 3-gene list generated to distinguish colon cancer from normal colon tissue with 90.32% accuracy [9]. CSRP1 expression was downregulated in 15 of 19 colorectal tumors; thus, it was suggested as a potential tumor suppressor in colorectal cancer (CRC) [10]. A recent study showed that a high expression of CSRP1 was associated with poor prognosis in The Cancer Genome Atlas (TCGA) colon adenocarcinoma (COAD) cohort [11]. However, evaluation of this gene as a prognostic marker has not yet been performed in independent cohorts.

In this study, the aim was to assess the biological and clinical parameters that are associated with CSRP1 expression in colon cancer. For this purpose, publicly available transcriptomic data from colon tumors and normal colon tissues were analyzed in parallel with previously generated molecular scoring and subgrouping methods that reflected the epithelial to mesenchymal transition (EMT) status, stromal fractions, and cancer-associated fibroblast (CAF) involvement. The findings herein showed that the CSRP1 expression was downregulated in colon cancer compared to normal colon tissue. Furthermore, the CSRP1 expression and methylation levels were negatively correlated in colon tumors. A high CSRP1 expression was associated with a mesenchymal and stroma-rich molecular profile and poor prognosis. Single-cell RNA sequencing (scRNAseq) and immunohistochemistry (IHC) data indicated that CSRP1 was expressed by epithelial and stromal cells in the tumor microenvironment.

## 2. Materials and methods

### 2.1. In silico analysis of the gene expression and methylation levels

Raw microarray-based transcriptomic data for primary COAD, GSE39582 (n = 566) and GSE17536 (n = 177) were downloaded from the Gene Expression Omnibus (GEO) database (https://www.ncbi.nlm.nih.gov/geo/query/acc.cgi) and RMA normalized [12,13]. An EMT score for samples in the GSE39582 and GSE17536 datasets were generated utilizing a previously published formula based on the expression of E-cadherin and vimentin (VIM) genes [14]. The EMT score has a range between −2 and 0 from the most epithelial to the most mesenchymal phenotype. Consensus molecular subtype (CMS) information was obtained from the Synapse web portal (www.synapse.org). Samples that could not be classified in any of the 4 CMSs were not used for comparison of the CSRP1 expression across the CMSs. A previously defined method, ESTIMATE, was utilized to obtain stromal scores inferring the fraction of stromal cells in the tumor samples [15]. ESTIMATE stromal scores for the GSE39582 dataset were generated using the ESTIMATE R package (University of Texas MD Anderson Cancer Center; https://bioinformatics.mdanderson.org/estimate/rpackage.html). ESTIMATE stromal scores based on the TCGA COAD RNAseq V2 data were downloaded (https://bioinformatics.mdanderson.org/estimate/disease.html). To define the tumor subgroups based on the CAF marker expression in the GSE39582 and GSE17536 datasets, a published list was used [16].

TCGA COAD primary tumor RNAseq data were downloaded from the National Cancer Institute Genomic Data Commons (GDC) portal (https://portal.gdc.cancer.gov/) in STAR-counts format. Formalin-fixed paraffin-embedded (FFPE) tissues were filtered out. For duplicate samples, the sample with the smaller total read count was excluded from the study. The remaining 451 unique tumors were used in the following steps. Genes with a count value of zero in more than 90% of the samples were removed from the count matrix. Counts were normalized using the DESeq2 package and log transformed [17]. 186 tumors had both the RNAseq-based gene expression data and ESTIMATE scores, which were used for the correlation analysis of the CSRP1 gene expression and stromal score in TCGA.

The linear correlation of CSRP1 expression and methylation status was studied via cBioPortal for Cancer Genomics (https://www.cbioportal.org/) [18,19]. Infinium Human Methylation 450K BeadChip and RNA expression (RNAseq v2) data in Colorectal Adenocarcinoma (TCGA, Firehose Legacy) datasets were used. Methylation beta and log(RSEM+1) values were utilized for the analysis of linear correlation. A comparison of the expression of CSRP1 in colorectal tumors and normal colon and rectum tissues was conducted via the Gene Expression Profiling Interactive Analysis (GEPIA) platform (https://gepia.cancer-pku.cn/) [20].

### 2.2. Log-rank-based prognostic analysis at multiple cut-off values

Cut-off-based evaluation of the prognostic relationships for the CSRP1 expression were conducted based on log-rank multiple cut-off graphs, as described previously [21]. Briefly, the patients were divided into 2 groups with a high and low expression of CSRP1 using each value of the CSRP1 expression as the cut-off value. Log-rank tests were performed based on the groups defined by each cut-off value. Log-rank p-values (y-axis) were plotted against all of the cut-off values (x-axis). Red and blue indicate the hazard ratio (HR) above and below 1, respectively, using the low expression group as a reference. The cut-off values with the lowest log-rank p-value within the interquartile range were used to define the groups with high and low expression for the Kaplan–Meier graphs.

### 2.3. scRNAseq data analysis

scRNAseq data of colorectal primary tumors were obtained from the GSE178318 dataset. Three patients who received no treatment were included in the analysis. An aligned raw gene expression matrix was downloaded from the GEO database and processed via the Seurat package in R Bioconductor [22]. Quality filtering was performed as follows: cells with less than 500 or more than 6000 expressed features, and cells with mitochondrial gene expression greater than 15% were filtered out. Doublets were detected and removed using the DoubletFinder package [23]. Next, 2000 variable features were determined using the FindVariableFeatures function. The uniform manifold approximation and projection method was used for dimension reduction. The list of cell-specific markers that was presented by Che et al. [24] was used to define the cellular clusters, with minor modifications. The NKG7 gene was added to the list of natural killer (NK) cell markers. The cell cluster that expressed CD68, CD163, CD14, and LYZ was named Myeloid cells.

### 2.4. Evaluation of the IHC-based CSRP1 expression

IHC data available in the Human Protein Atlas were used [25, 26]. IHC-based staining for CSRP1 (clone: HPA045617) was evaluated in colon tumors. The consensus dataset of the RNA expression obtained from healthy tissues was utilized for the assessment of the CSRP1 expression.

### 2.5. Statistical analysis

Kaplan–Meier graphs were generated using Graphpad Prism 8 (San Diego, CA, USA). One-way analysis of variance (ANOVA), Tukey’s multiple comparison tests, unpaired t tests, and Pearson correlation analysis were performed using Graphpad Prism 8. Cox univariate and multivariate regression analysis (MVA) were performed using IBM SPSS Statistics for Windows 23.0 (IBM Corp., Armonk, NY, USA. Patients with nonzero survival data were included in these analyses. Documented recurrence (0 = no, 1 = yes) was used as status for DFS.

## 3. Results

### 3.1. CSRP1 expression in malignant and benign tissues

**Figure 1:**
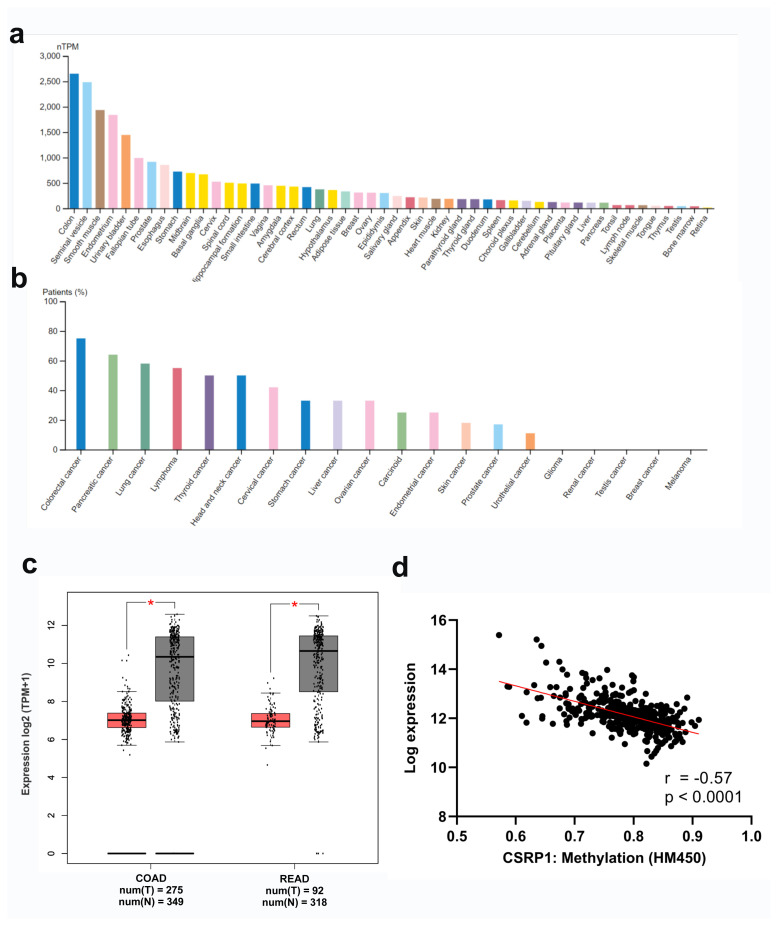
Expression and methylation of CSRP1 in the healthy and malignant tissues. RNAseq-based expression of CSRP1 in healthy tissues within the consensus dataset of Human Protein Atlas (a) and the percentage of patients with a moderate or high protein level of CSRP1 expression across the available cancer types (b) are shown. The expression of CSRP1 in colon and rectal tumors (TCGA) compared to a normal colon and rectum (TCGA+GTEx) were plotted using the GEPIA web server (c). * p < 0.05. Correlation of CSRP1 expression and methylation data obtained from cBioportal database (n = 372) (d). Pearson r- and p-values are indicated.

The CSRP1 gene expression was evaluated in various cancer types and healthy tissues using publicly available datasets. Based on the consensus RNAseq dataset of the Human Protein Atlas, the expression of CSRP1 was the highest in colon tissue among the 54 healthy tissues available (Figure 1a). At the protein level, measured via IHC, the percentage of patients with moderate or high expression was the highest in CRC among the 20 cancer types (Figure 1b). When the expression of CSRP1 was analyzed via the GEPIA web tool, a significantly lower expression of CSRP1 was noted in both colon and rectal tumors (TCGA) compared to normal colon and rectum tissue (TCGA+GTEx) (log fold change >3, p < 0.05) (Figure 1c). These data showed that the CSRP1 gene was expressed at high levels in both the malignant and normal colonic tissues, and its expression was lower in colon tumors compared to normal colon tissue.

DNA methylation is one of the main epigenetic mechanisms of regulation of gene expression levels and CSRP1 was shown to be inactivated via aberrant methylation in HCC [7]. To understand whether DNA methylation could be a potential mechanism that is involved in the regulation of CSRP1 expression in CRC, publicly available CSRP1 methylation and expression data from TCGA colorectal adenocarcinoma cohort were analyzed. Indeed, the methylation and expression levels were strongly negatively correlated (r = −0.57, p < 0.0001) for CSRP1, suggesting that DNA methylation may have a role in the transcriptional regulation of this gene (Figure 1d).

### 3.2. Association of the CSRP1 expression with prognosis and clinicopathological parameters

**Figure 2:**
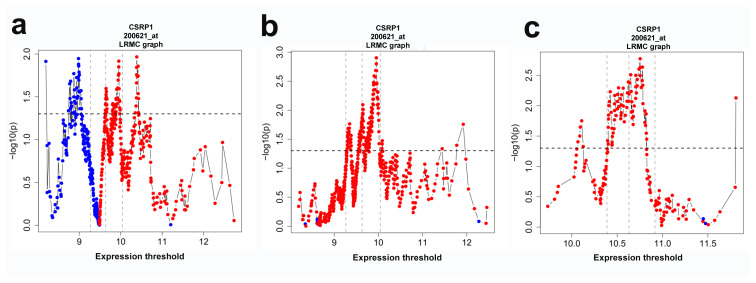
Log-rank multiple cut-off graphs for the CSRP1 expression. Log-rank test results are plotted at all cut-off values: the graphic indicates the log-rank values (shown as dots) obtained at every possible cut-off for CSRP1 in GSE39582 (a–b) and GSE17536 (c) for OS (a), RFS (b) and DSS (c). Red and blue indicate that high CSRP1 expression is associated with poor (HR > 1) and good prognosis (HR < 1), respectively. Horizontal dotted line p = 0.05. Vertical dotted lines: (from left to right) first 25th percentile, median, and 75th percentile.

**Figure 3:**
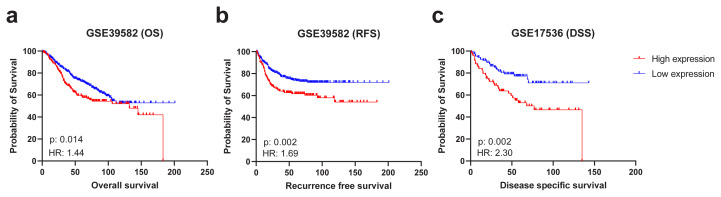
Kaplan–Meier plots for the CSRP1 expression in GSE39582 and GSE17536. Prognostic comparison in GSE39582 and GSE17536 are shown in (a), (b), and (c), respectively. OS (a) and RFS (b) were used as clinical end-points for GSE39582. DSS was used for GSE17536 (c). Hazard ratios and log-rank p-values are indicated.

A recent study identified that a high CSRP1 expression was an independent marker of poor prognosis (HR = 1.895, 95% CI: 1.078–3.330, p = 0.026) in TCGA COAD dataset [11]. In order to evaluate the robustness of CSRP1 as a prognostic marker across multiple cohorts, independent transcriptomic datasets were analyzed. A high expression of CSRP1 was significantly associated with poor prognosis, consistently, at multiple cut-off values within the interquartile range of the CSRP1 expression in the GSE39582 (Figures 2a and 2b) and GSE17536 (Figure 2c) datasets. A high CSRP1 expression was associated significantly (p < 0.05) with overall survival (OS) (HR = 1.442, 95% CI: 1.054–1.973) (Figure 3a) and recurrence-free survival (RFS) (HR = 1.691, 95% CI: 1.181–2.422) in the GSE39582 dataset (Figure 3b), and disease-specific survival (DSS) (HR = 2.295, 95% CI: 1.331–3.959) in the GSE17536 dataset (Figure 3c). No significant association was noted with disease free survival in the GSE17536 dataset. Overall, a high CSRP1 expression was associated with poor prognosis in 2 independent cohorts when tested with 3 different measures of clinical outcome (OS, RFS, and DSS).

**Figure 4:**
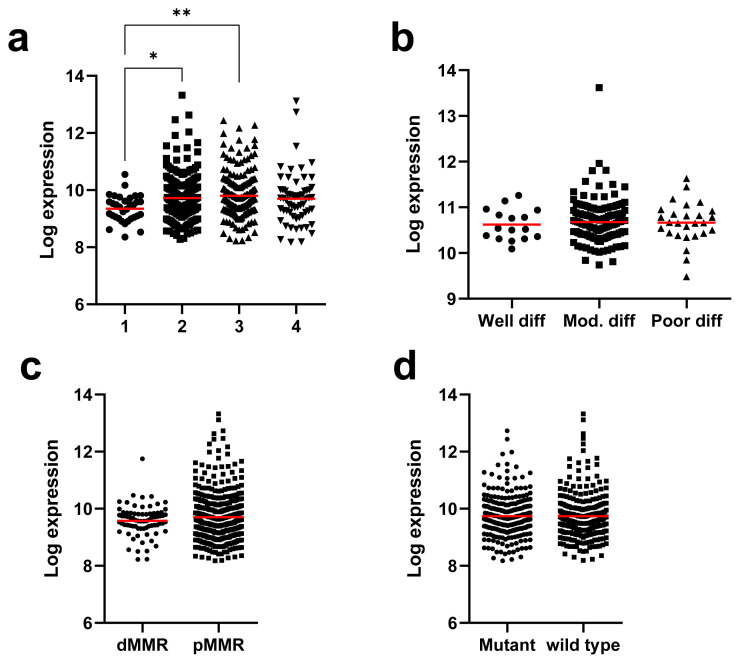
Expression of CSRP1 in colon tumors analyzed within subgroups categorized based on clinicopathological parameters. CSRP1 expression across the TNM stage (a), grade (b), and MSI status (c) and KRAS-BRAF mutation status (d) are plotted. Mutant: with KRAS or BRAF mutation, wild type: wild type for both genes. dMMR: deficient mismatch repair, pMMR: proficient mismatch repair, Mod: moderately, diff: differentiated. The GSE39582 (a,c,d) and GSE17536 (b) datasets were used. Tukey’s test for multiple comparison p-value; * p < 0.05, ** p < 0.01.

To assess whether CSRP1 is an independent prognostic marker, MVA was performed with the confounding factors identified previously in the GSE39582 dataset [21]. The results revealed that the CSRP1 expression was a significant predictor of poor prognosis independent of the microsatellite instability (MSI) status, TNM stage, and KRAS-BRAF mutation status (Table 1). Although the differences between the mean expression levels were minor (the mean expression was 9.35, 9.72, 9.80, and 9.69 for stages 1, 2, 3, and 4, respectively), the CSRP1 expression was significantly lower in stage 1 compared to stages 2 and 3 (Figure 4a). When the CSRP1 expression was compared across tumors with varying clinical characteristics, there was no significant difference between tumors with different grades (Figure 4b), the MSI status (Figure 4c), and tumors with and without the KRAS or BRAF mutation (Figure 4d).

### 3.3. Association of the CSRP1 expression with a mesenchymal, stroma rich molecular profile

**Figure 5:**
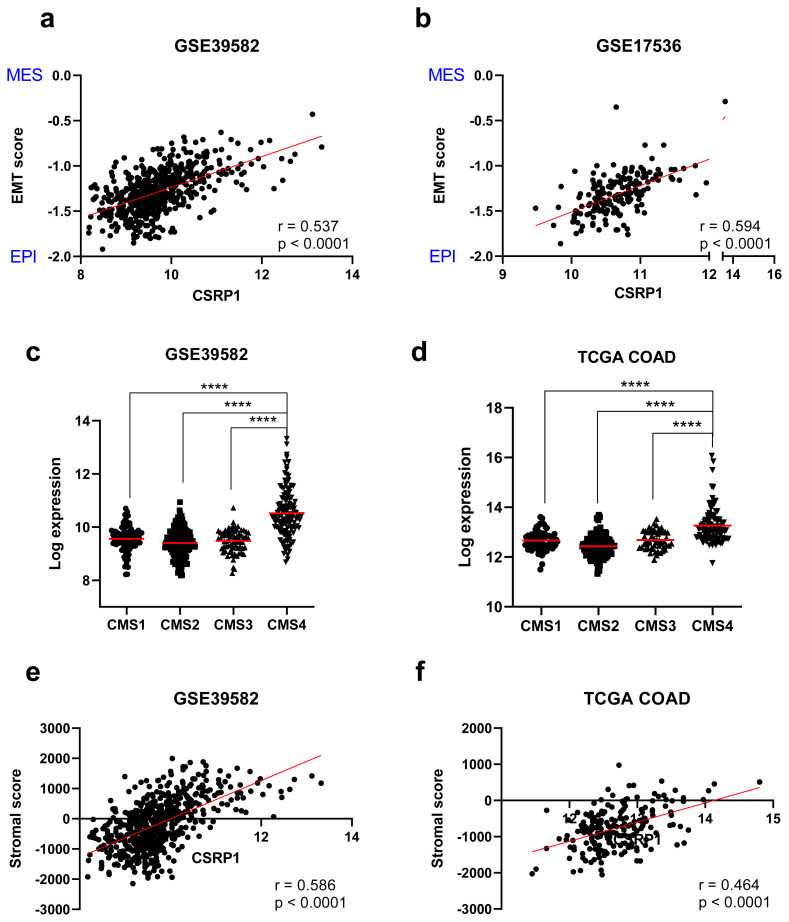
Higher CSRP1 expression in CMS4, mesenchymal, and stroma-rich tumors. Correlation of CSRP1 expression and EMT score is shown for the GSE39582 (a) and GSE17536 (b) datasets. EPI: epithelial, MES: mesenchymal. CSRP1 expression in the CMSs for the GSE39582 (c) and TCGA COAD (d) datasets are plotted. Correlation of CSRP1 and stromal score is plotted for the GSE39582 (e) and TCGA COAD (f) cohorts. Pearson r- and p-values are indicated. **** p < 0.0001.

Previous studies have indicated a strong association between EMT and the ability of cancer cells to invade other tissues and it is a key mechanism contributing to the progression of CRC [27]. As a high CSRP1 expression was associated with a shorter RFS, next, the relationships between the expression of CSRP1 and mesenchymal markers (SNAI1, SNAI2, ZEB1, ZEB2, TWIST1, TWIST2, and VIM) were studied. The CSRP1 expression had a significant positive correlation with all 7 of the mesenchymal markers tested (p < 0.001) in both the GSE39582 and TCGA COAD datasets (Table 2). The mean of the 2 r-values from these datasets were above 0.40 for 4 of the genes, ZEB1, ZEB2, TWIST2, and VIM (Table 2), indicating a strong correlation of the CSRP1 expression with the mesenchymal markers. These findings were confirmed by applying a previously published EMT scoring method (see the Materials and methods section) in the GSE39582 and GSE17536 datasets. The CSRP1 expression showed a significant positive correlation with the EMT score (r > 0.50, p < 0.0001) (Figures 5a and 5b). Overall, these data revealed that the CSRP1 expression was correlated with the mesenchymal gene expression, suggesting an association with relatively mesenchymal subtypes of colon cancer.

The Colorectal Cancer Subtyping Consortium defined CMS4-type tumors as a mesenchymal tumor subtype that harbors prominent transforming growth factor β activation, and angiogenesis [28]. Therefore, the CSRP1 expression was next evaluated among the CMSs. The CMS4-type tumors had a significantly higher CSRP1 expression when compared to the other 3 types, CMS1, CMS2, and CMS3, in both the GSE39582 (Figure 5c) and TCGA COAD datasets (Figure 5d). The CMS4-type was also associated with a higher stromal invasion [28]. Accordingly, a previously developed method, ESTIMATE, was utilized to assess whether the stromal fractions in the tumor microenvironment are correlated with the CSRP1 expression. The stromal scores generated by ESTIMATE, inferring the presence of stroma in tumor tissue, were significantly correlated with the CSRP1 expression in both the GSE39582 (Figure 5e) and TCGA COAD datasets (r > 0.45, p < 0.0001) (Figure 5f). Taken together, these findings indicated that tumors with a high CSRP1 expression had a stroma-rich profile that overlapped, to a large extent, with a CMS4 phenotype.

### 3.4. Epithelial cells and CAFs express CSRP1 in the colon tumor microenvironment

**Figure 6:**
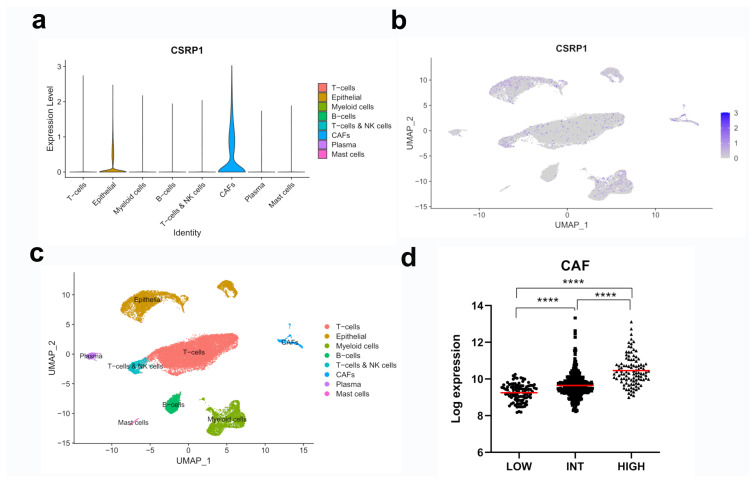
CSRP1 is expressed in the epithelial cells and CAFs in primary colorectal tumors. scRNAseq (GSE178318) data were processed and expression of CSRP1 is shown via a violin plot for 8 cell clusters (a). Feature plots depicting the CSRP1 expression (b) and cell clusters (c) are indicated. The intensity of the purple indicates the level of expression. The CSRP1 expression is shown in the tumor subgroups with low, intermediate (INT), and high CAF marker expression in GSE39582 (d). **** Unpaired t-test p < 0.0001.

In order to identify the cell types that express CSRP1, a scRNAseq dataset (GSE178318) of colorectal tumors was analyzed. The CSRP1 expression was detected mainly in epithelial cells and CAFs, but very low or no expression was noted in cell clusters that included T cells, myeloid cells, B cells, NK cells, plasma, and mast cells (Figures 6a–6c). Among all of the cell types, CAFs expressed CSRP1 at the highest level, which is in line with the higher expression of CSRP1 in tumors with higher stromal fractions. Furthermore, the CSRP1 expression was analyzed within previously defined CRC subgroups based on the expression of CAF markers [16]. The analysis showed that tumors with a higher CAF marker expression (high group) had increased CSRP1 expression, which gradually decreased in tumors with intermediate and low CAF marker expression (Figure 6d) (p < 0.0001).

**Figure 7:**
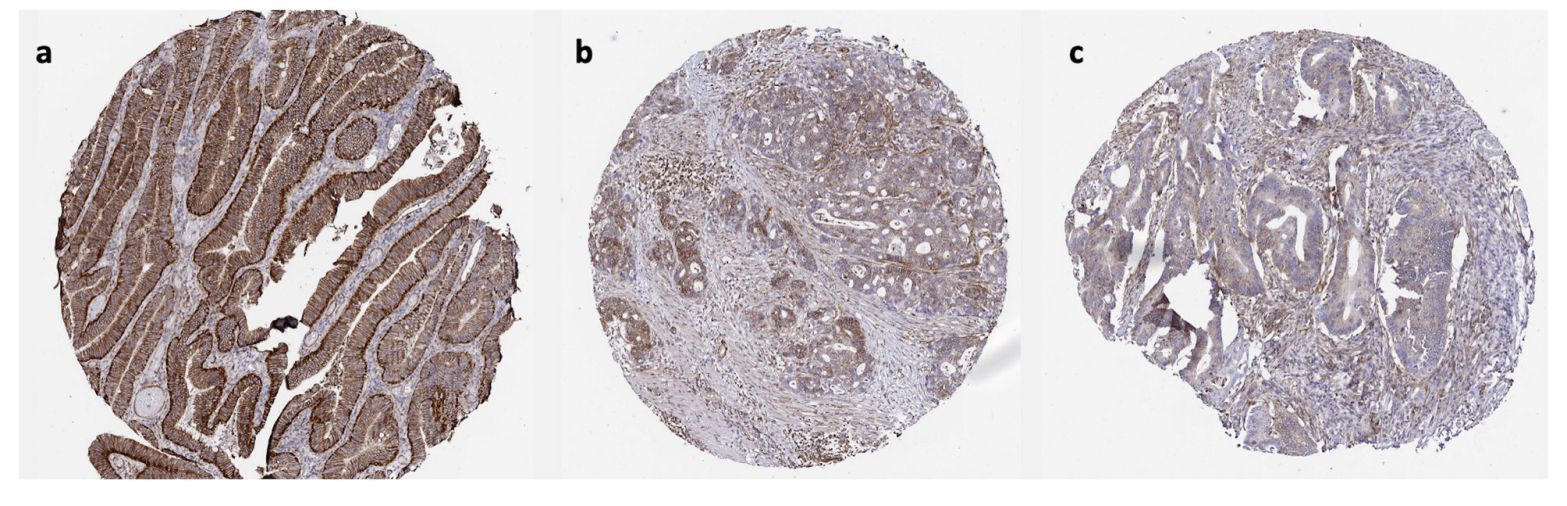
IHC**-**based staining for CSRP1. Representative cases with CSRP1 expression primarily in the epithelial neoplastic (a) and stromal (c) cells, and cases with expression in both cell types (b).

Evaluation of the IHC-based expression of CSRP1 in colon tumors available in the Human Protein Atlas showed that 4, 5, and 2 cases had high, intermediate, and low expression of CSRP1, respectively (https://www.proteinatlas.org/ENSG00000159176-CSRP1/pathology/colorectal+cancer). No expression was detected for 1 patient. These data indicate that CSRP1 was, indeed, expressed at the protein level in the colon tumors, and the level of expression varied among the cases. The subcellular localization was in cytoplasmic/membranous regions for all of the patients. The CSRP1 expression was noted in both epithelial neoplastic cells (Figures 7a and 7b) and spindle stromal cells (mostly fibroblasts) (Figures 7b and 7c), supporting the presence of CSRP1 expression in epithelial cells and CAFs in the scRNAseq data.

## 4. Discussion

CSRP1, a smooth muscle marker that has a role in actin filament bundling, has been previously implicated in multiple cancer types. A lower expression of CSRP1 in tumors compared to normal colon tissue was reported in sporadic CRC cases by Zhou et al., and thus CSRP1 was suggested as a putative tumor suppressor [10]. Consistently, the data herein showed that its expression was significantly lower in colorectal tumors compared to normal colon and rectum tissues. In HCC, the CSRP1 expression was suppressed via promoter methylation [7], and, in line with that, the data herein showed a significant negative correlation between RNAseq-based expression and the methylation of the CSRP1 gene in colon cancer. These data suggested that the CSRP1 expression may also be regulated via promoter methylation in CRC. However, the tumor-suppressor-like expression and methylation changes of CSRP1 need further investigation since its relationship with poor prognosis was shown. Although the regulation of CSRP1 expression has not yet been fully elucidated, TGF-β1 was shown to play a role in inducing CSRP1 expression through the Smad and nonconventional p38 MAPK signaling pathways [6], which may also be involved in regulating the CSRP1 expression during malignant transformation.

Analysis of the prognostic relationships of CSRP1 in the TCGA COAD dataset previously showed that its expression was associated with unfavorable prognosis [11]. However, this result has not yet been evaluated in independent cohorts. As reevaluation of the data in a completely different set of samples is crucial to confirm the validity of the putative molecular markers [29], the current study revealed that a high CSRP1 expression was consistently associated with worse prognosis in independent cohorts. A prognostic analysis with multiple different measures of clinical outcome (OS, RFS, and DSS) further indicated that the CSRP1 expression is linked with both the disease aggressiveness and time to death. The fact that these relationships were shown based on transcriptomic data generated from multiple technologies (RNAseq and microarray) further supports the robustness and power of CSRP1 as a prognostic marker. The prognostic relationships of CSRP1 have been studied in several different cancer types. CSRP1 was included in a gene signature that identified a high-risk subgroup of acute myeloid leukemia with worse prognosis [30]. In contrast, in prostate cancer, it was reported that a high expression of CSRP1 was associated with longer DFS [8]. Therefore, CSRP1 may be related to different processes in cancers of different tissues of origin. Alternatively, the expression of CSRP1 in the tumor microenvironment of these tissues may be a critical factor underlying its relationships with disease progression. A recent study showed that the knockdown of CSRP1 in colon cancer cell lines caused a reduction in cell proliferation and migration [11]. Several studies have revealed the roles of CSRP1 also in cellular contractility and movement. A reduction in cellular contractility was noted in human fibroblasts following CSRP1 silencing [6]. The involvement of CSRP1 in cellular movement has been shown in zebrafish, as the knockdown of CSRP1 caused abnormal convergent extension cell movement, resulting in severe deformities in midline structures [31]. Overall, these data highlight the relationship of the CSRP1 expression with aggressive cell behavior in specific tissues and malignancies.

EMT is one of the major mechanisms involved in cancer progression and poor clinical outcomes [32]. Thus, linear relationships between the expression of CSRP1 and EMT markers were investigated to explore the potential underlying molecular mechanisms that can explain the relation of high CSRP1 expression and poor prognosis. Positive correlations between the expression of CSRP1 and EMT markers were consistent when tested in independent cohorts and with different technologies measuring transcriptomic levels (microarray, RNAseq). Therefore, CSRP1 may be related to cancer progression via various mechanisms related to proliferation, EMT, and cell migration.

In silico analyses of bulk transcriptomic data from colon tumors showed that tumors with a high CSRP1 expression had a stroma-rich molecular profile that overlapped, to a large extent, with a CMS4 phenotype. In line with that, the data herein revealed the expression of CSRP1 mainly in epithelial cells and CAFs in the tumor microenvironment. CSRP1 was previously shown to be regulated by TGF-β1, inducing myofibroblast differentiation. Furthermore, the CSRP1 expression was significantly higher in idiopathic pulmonary fibrosis compared to control lung tissue [6]. These data suggest that CSRP1 may have roles in cancer progression, not only via its roles in cancer cells, but also through its potential roles in stromal cells, likely fibroblasts, in the tumor microenvironment.

In short, the CSRP1 expression was associated with a more mesenchymal, stroma-rich molecular profile and poor prognosis in colon cancer. With further large-scale validation, the CSRP1 gene might be shown to have the potential to contribute to patient stratification in colon cancer. Future studies investigating the role of CSRP1 in CRC stroma may pave the way to the evaluation of this gene as a potential therapeutic target.

## Figures and Tables

**Table 1:** Multivariate analysis in GSE39582 (RFS).

				95% CI for HR		
	No. of patients	Percentage	HR	Lower	Upper	p-value
MSI status						
pMMR (ref.)	444	78.45%	0.43	0.23	0.82	**0.011**
dMMR	75	13.30%				
NA	47	8.30%				
TNM stage*						
0	4	0.70%	1.66	1.27	2.16	**<0.001**
1	33	5.83%				
2	264	46.64%				
3	205	36.20%				
4	60	10.60%				
KRAS or BRAF mutation						
Mutant**	268	47.35%	1.37	0.95	1.99	0.095
Wild type (ref.)	255	45.05%				
NA	43	7.60%				
CSRP1 expression						
High	170	30.04%	1.94	1.35	2.80	**<0.001**
Low (ref.)	396	69.96%				

* Treated as a continuous variable (1, 2, 3, and 4),

** mutant for KRAS or BRAF.

Reference groups are indicated (ref.) for the categorical variables. pMMR: proficient mismatch repair, dMMR: deficient mismatch repair, HR: hazard ratio, CI: confidence interval.

**Table 2:** Linear correlation of CSRP1 expression with the expression of EMT markers.

	GSE39582	TCGA COAD	
	(n = 566)	(n = 451)	Mean r*
SNAI1	0.183	0.223	0.203
SNAI2	0.385	0.357	0.371
ZEB1	0.496	0.478	0.487
ZEB2	0.470	0.361	0.416
TWIST1	0.422	0.363	0.392
TWIST2	0.598	0.431	0.514
VIM	0.557	0.495	0.526

Pearson r-values are indicated. All correlations were significant (p < 0.001).

* Average of the 2 r-values from GSE39582 and TCGA COAD.
